# Seasonal impact of diurnal temperature range on intracerebral hemorrhage in middle-aged and elderly people in central China

**DOI:** 10.4178/epih.e2024053

**Published:** 2024-06-11

**Authors:** Shiwen Wang, Jinyu Yin, Hao Zhou, Jingmin Lai, Guizhen Xiao, Zhuoya Tong, Jing Deng, Fang Yang, Qianshan Shi, jingcheng shi

**Affiliations:** 1 Department of Epidemiology and Medical Statistics, Xiangya School of Public Health, Central South University, Changsha, China; 2 Discipline Construction Office of XiangYa Hospital, Central South University, Changsha, China; 3 Information Statistics Center of Health Commission of Hunan Province, Changsha, China

**Keywords:** Diurnal temperature range, Intracerebral hemorrhage, Middle aged, Elderly, Hospitalization, Distributed lag nonlinear model, Seasons

## Abstract

**OBJECTIVES:**

This study investigated the seasonal impact of diurnal temperature range (DTR) on hospitalization rates for intracerebral hemorrhage (ICH) in middle-aged and elderly adults.

**METHODS:**

We collected data on the DTR and hospitalization records of ≥45-year-old patients with ICH in 2019 in Hunan Province, central China. Time-series analyses were performed using a distributed lag non-linear model.

**RESULTS:**

Overall, 54,690 hospitalizations for ICH were recorded. DTR showed a non-linear relationship with ICH hospitalization in both middle-aged and elderly populations (45-59 and ≥60 years, respectively). During spring, a low DTR coupled with persistently low temperatures increased ICH risk in both age groups, while a high DTR was associated with an increased risk in the middle-aged group only (relative risk [RR], 1.24; 95% confidence interval [CI], 1.21 to 1.27). In the summer, a low DTR combined with persistently high temperatures was linked to a higher risk exclusively in the middle-aged group. A high DTR in the autumn was correlated with increased risk in both age groups. In winter, either a low DTR with a continuously low temperature or a high DTR elevated the risk solely in the elderly population (RR, 1.37; 95% CI, 1.00 to 1.69). In the elderly group, the impact of DTR on hospitalization risk manifested within a 5-day period.

**CONCLUSIONS:**

The impact of DTR on ICH hospitalization risk differed significantly across seasons and between age groups. Elderly individuals demonstrated greater sensitivity to the impact of DTR. Weather forecasting services should emphasize DTR values, and interventions targeting sensitive populations are needed.

## GRAPHICAL ABSTRACT

[Fig f4-epih-46-e2024053]

## Key Message

In a large-scale, study of 54,690 hospitalizations for intracerebral hemorrhage (ICH) from China, we identified the seasonal impact of diurnal temperature range (DTR) on hospitalization rates for intracerebral hemorrhage (ICH) in middle aged and elderly adults. Specifically, the impact of DTR on ICH hospitalization risk differed significantly across seasons and between age groups. Elderly individuals demonstrated greater sensitivity to the impact of DTR. The findings of this study could be instrumental in the prevention and control of ICH.

## INTRODUCTION

Intracerebral hemorrhage (ICH) is a clinical syndrome characterized by the non-traumatic rupture of blood vessels within the brain, resulting in the accumulation of blood in the brain parenchyma. This can lead to cognitive impairment, mental and emotional disorders, and other symptoms [1,2]. ICH is known for its rapid onset and potentially serious consequences, with the highest mortality rate among all stroke types [3,4]. According to 2019 data on the global burden of disease, the incidence of ICH in China is between 49.4 and 71.9 per 100,000 people. While this rate is the highest in the world, it represents a decrease of 2.1% compared to 1990. However, the mortality rate has increased by 14.4%, making ICH the stroke subtype with the greatest mortality in China [5,6]. ICH is particularly prevalent among middle-aged and elderly individuals [7,8]. In 2018, the total direct medical costs for ICH in China amounted to 20.512 billion yuan per year, which accounted for 3.91% of the nation’s total medical expenses—an enormous financial burden [9-11]. Therefore, the early prevention of ICH is crucial.

The diurnal temperature range (DTR) reflects the variation in temperature within a single day and is defined as the difference between the daily maximum and minimum temperatures [12]. It represents a key meteorological indicator of global climate change. As with temperature, exposure to DTR is unavoidable, and its link to the development of ICH is an important public health issue [13-18]. Studies have examined the association between ICH mortality and DTR, suggesting that a higher DTR may elevate the risk of mortality in patients with ICH [19-22]. Accordingly, most research has concentrated on mortality outcomes. However, the growing burden and costs associated with stroke care underscore the pressing need for effective prevention strategies [23-28]. Nonetheless, the evidence pertaining to the relationship between DTR and the onset of or hospitalization for ICH remains ambiguous, largely due to inadequate control for confounding factors such as ambient temperature, limited geographic scope of studies, and insufficient consideration of varying temperature exposures and physical adaptations among subgroups [29-33]. The impact of DTR on hospitalizations for ICH warrants further study [34,35], as do the health implications of DTR across different seasons and subpopulations [36-38].

Hunan Province, a representative region of central China, spans from 108°47´ to 114°15´ east longitude and from 24°38´ to 30°08´ north latitude. In Changsha, the capital city of Hunan, the incidence of ICH is 131.0 per 100,000 persons, which is substantially higher than the global average [39]. The region experiences 4 distinct seasons, with sufficient heat, concentrated rainfall, variable spring temperatures, dry summers and autumns, a brief but intense cold period, and an extended period of summer heat. These climatic conditions provide Hunan with a geographic advantage for studying the effects of DTR on ICH hospitalization rates [40].

We employed a distributed lag non-linear model to examine the influence of DTR on ICH. Our analysis centered on DTR as the primary exposure, incorporating its interaction with temperature in different seasons as well as additional variables, such as social factors, within a population of middle-aged and elderly individuals in central China.

## MATERIALS AND METHODS

### Data collection

The study included a total of 54,690 patients aged 45 years or older who were admitted to secondary (554 in total) or tertiary (103 in total) hospitals with a primary diagnosis of ICH (I61) between January 1, 2019 and December 31, 2019. These data were obtained from the direct statistical reporting system of the Hunan Health Commission. The geographic distribution of the secondary and tertiary hospitals is depicted in Supplementary Material 1. Daily meteorological data were obtained from the China Meteorological Data Network (https://data.cma.cn/) and categorized into 4 seasons: spring (March to May), summer (June to August), autumn (September to November), and winter (December to February) [12]. Daily air quality data were sourced from the National Air Pollution Monitoring System (http://envi.ckcest.cn/environment/data_Integration/data_Integration.jsp). Information on the population, economy, and health resources for each district/county in Hunan Province for the year 2019 was extracted from the Hunan Statistical Yearbook 2020 (http://tjj.hunan.gov.cn/).

**Figure 1.**
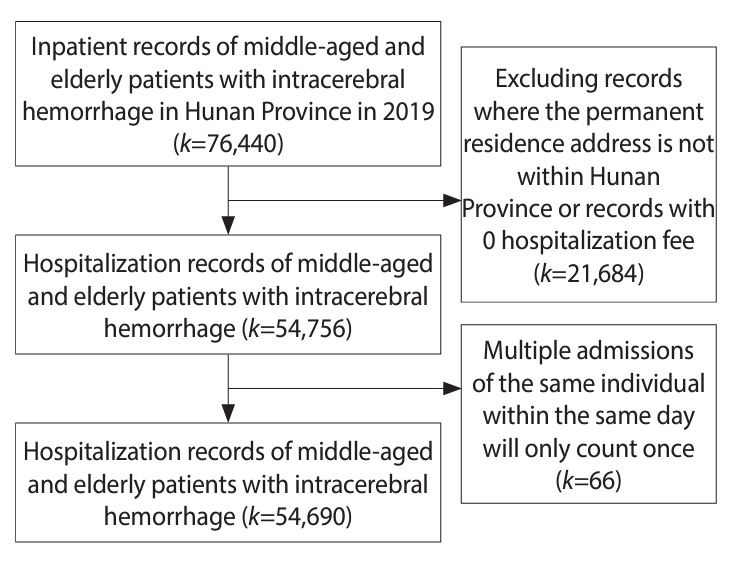
A flowchart of the data collection process. *k*, number of hospitalization records.

For admitted patients, the inclusion criteria encompassed: (1) a hospitalization date between January 1, 2019 and December 31, 2019; (2) a primary discharge diagnosis of ICH, under the International Classification of Diseases, 10th revision (code I61); (3) an age of 45 years or older (patients 45 to 59 years old were categorized as middle-aged, while those 60 years old or older were considered elderly) [41]; and (4) a minimum hospital stay of 1 day. The exclusion criteria were: (1) an address that was either unclear or not located in Hunan Province and (2) ICH resulting from trauma. The data collection process is illustrated in Figure 1.

### Exposure assessment

Hunan Province is equipped with 29 environmental/meteorological monitoring stations and 71 air quality monitoring stations. To obtain daily average meteorological factors and air pollution levels for each county or district, spatiotemporal Kriging interpolation analysis was employed. This method was used to supplement the daily meteorological and air quality data for each district and county of Hunan Province for the year 2019.

Data checking and screening included several processes. (1) Logical verification involved removing admissions with clear logical errors and duplications. For example, individuals with hospitalization costs of 0 were excluded, and multiple admissions of a patient on the same day were counted as a single admission. Otherwise, admissions were treated as separate, independent events. (2) Regarding missing data, admissions lacking essential information (such as present residence, household registration address, or discharge diagnosis information) were omitted from the analysis. (3) The Q-test was employed to identify univariate outliers. Any outlier data identified were then verified and either removed or adjusted accordingly.

### Statistical analysis

In this study, percentiles were used to describe meteorological factors and air pollutant levels (Supplementary Material 2). We tested the correlation between air pollutant concentrations and meteorological factors (Supplementary Material 3). Any pair of variables with a correlation coefficient greater than 0.8 was considered strongly correlated, and a single variable was selected for inclusion in the model according to the clinical significance and goodness-of-fit. Additionally, we conducted an over-dispersion test; this yielded a p-value of less than 0.05, indicating that the data were over-dispersed. Consequently, a quasi-Poisson regression was employed to fit the time-series model.

The selection of independent variables was informed by a literature review and a priori knowledge from experts about factors known to be associated with time, as well as their presence in the dataset. The controlled potential covariates included [42-45]: (1) environmental factors: diurnal mean temperature (denoted in the model as “temp”), relative humidity (“relahumidity”), particulate matter with diameters of 2.5 micrometers and smaller (PM_2.5_), particulate matter with diameters of 10 micrometers and smaller (PM_10_), nitrogen dioxide (NO_2_), sulfur dioxide (SO_2_), carbon monoxide (CO), and ozone (O_3_); and (2) social factors: gross regional product (“GDP”), average population (“popu”), the number of medical institutions in each district/county in 2019 (“hospitals”), day of the week (“DOW”), and public holidays (“holiday”).

Based on the data presented, we applied 2 sets of time-series quasi-Poisson models to estimate the associations between DTR and ICH hospitalization, utilizing the distributed lag non-linear model framework. In the first time-series quasi-Poisson model analysis—model (1)—we adjusted for seasonality to estimate the overall impact of DTR on hospitalization for ICH among middle-aged and elderly populations. Correlation analysis indicated that PM_2.5_ was strongly correlated with PM_10_ (r_s_= 0.83, p<0.05). Given the practical significance and goodness-of-fit, PM_10_ was excluded from this analysis. The initial model was formulated as follows:


(1) $$\mathrm{Log}(E(Y_{t,i}))=\alpha +\beta DTR_{t,i,l}+ns(temp_{t,i},\;df=3)+\;ns({\mathrm{relahumidity}}_{t,i},\;df=3)+\nu GDP_{i}+\tau popu_{i}+\vartheta {\mathrm{hospitals}}_{i}+\;DOW+\mathrm{holiday}+ns(PM2.5_{t,i},\;df=3)+ns(NO2_{t,i},\;df=3)+\;ns(SO2_{t,i},\;df=3)+ns(CO_{t,i},\;df=3)+ns(O3_{t,i},\;df=3)+\;ns("\mathrm{time}",\;df=3\times 4)$$

Here, *α* represents the intercept, *β* symbolizes the log-relative risk of hospitalization associated with a unit increase in DTR, *t* denotes the day of the year (from 1 to 365), *i* is the code number for the district/county, and *l* represents the lag in days. E(*Y_t,i_*) indicates the expected count of hospitalizations in area *i* on day *t*. *DTR_t,i,l_* is a cross-basis matrix that assesses the non-linear and lag effects of DTR on ICH [46]. *Time* denotes long-term effects; accordingly, we set the degrees of freedom (df) to 12 [47]. Consistent with previous research, our primary model incorporated a natural cubic spline of calendar days with 12 df per year to adjust for seasonality and long-term trends. We tested different df for time, ranging from 4 to 16, and found that 12 yielded the lowest Akaike information criterion (AIC). The definitions of the other variables are available in the previous text. The nodes of the exposure-response relationship were established at the 25th, 50th, and 75th percentiles. Based on the AIC, we determined the maximum lag days to be 14. The environmental variables, including relative humidity, diurnal mean temperature, PM_2.5_, PM_10_, NO_2_, SO_2_, CO, and O_3_, were modeled using natural cubic spline functions (ns), with the df set at 3.

In the second time-series quasi-Poisson model, labeled model (2), we conducted separate analyses for each age group in the different seasons. Specifically, we calculated the relative risk (RR) by using the DTR associated with the lowest risk as a reference to estimate the effect of DTR during each season. We constructed models individually for the 2 age groups in the 4 seasons. The methodology was as follows: (1) the median DTR of each season was used as the initial reference for model fitting; (2) the DTR corresponding to the lowest RR was identified for each season and served as the updated reference; and (3) the model was refitted using this updated reference DTR to estimate the RR.

The model for each season was expressed as follows. The meanings of the variables are consistent with model (1).


(2) $$\mathrm{Log}(E(Y_{t,i}))=\alpha +\beta DTR_{t,i,l}+ns(temp_{t,i},\;df=3)+\;ns({\mathrm{relahumidity}}_{t,i},\;df=3)+\nu GDP_{i}+\tau popu_{i}+\vartheta {\mathrm{hospitals}}_{i}+\;DOW+\mathrm{holiday}+ns(PM2.5_{t,i},\;df=3)+ns(NO2_{t,i},\;df=3)+\;ns(SO2_{t,i},\;df=3)+ns(CO_{t,i},\;df=3)+ns(O3_{t,i},\;df=3)$$

Unless otherwise specified, all p-values represent 2-sided probabilities, with the significance level set at *α*=0.05. To ensure the robustness of our main results, we conducted a sensitivity analysis by varying the df (from 3 to 5) and the maximum lag days (from 14 to 21) for DTR. Additionally, sensitivity analyses were performed by altering the number of times of admission (1, 2, > 2). All analyses were conducted using the “dlnm” package (version 2.4.7) in R version 4.2.1 (R Foundation for Statistical Computing, Vienna, Austria). The names, identification numbers, and telephone numbers of patients were desensitized during the data collection phase. We adhered to data confidentiality principles throughout the study.

### Ethics statement

This study is an ecological analysis of hospitalization records. Patient names, identification numbers, and telephone numbers were desensitized during the data collection phase. Data confidentiality principles were followed throughout the study. Approval for this study was granted by the Xiangya School of Public Health, Central South University (approval No. XYGW-2019-040).

## RESULTS

Table 1 presents the daily hospital admissions for middle-aged and elderly individuals with ICH in Hunan Province during the year 2019. The total number of admissions was 54,690, corresponding to a hospitalization rate of 0.24%. Of these admissions, 16,824 were middle-aged patients, representing 44.43% of the total and exhibiting a hospitalization rate of 0.13%. The remaining 37,866 patients were elderly, comprising 55.57% of the admissions and representing a hospitalization rate of 0.40%.

The ICH hospitalization rate and the number of admissions were higher in the elderly than in the middle-aged population. Supplementary Material 4 presents the average length of ICH hospitalization in Hunan in 2019. Male and elderly individuals experienced longer hospital stays, with the duration of ICH hospitalizations being longest during the winter for all groups.

Table 2 and Supplementary Material 5 indicate that the highest number of ICH hospitalizations among the middle-aged and elderly populations occurred in January, with March and November also displaying high admission numbers. Hospital admissions were more frequent in spring and winter and less frequent in summer. Additionally, the monthly variation in admission person-times was greater in the elderly group compared to the middle-aged group, with a range of 2,154 compared to 875, respectively.

Supplementary Material 2 presents the daily meteorological and air quality data for 122 districts/counties in Hunan Province for 2019. Supplementary Material 3 displays the correlation results between these variables. Correlation analysis indicated that DTR was positively correlated with daily average temperature (r_s_=0.34, p < 0.05). Additionally, PM_2.5_ was strongly correlated with PM_10_ (r_s_=0.83, p<0.05). Due to considerations of practical significance and goodness-of-fit, PM_10_ was excluded from this analysis.

Supplementary Material 6 presents a line graph depicting the monthly average temperature and DTR in Hunan Province for 2019. The DTR trend exhibited a bimodal pattern, with peaks in April and October.

**Figure 2.**
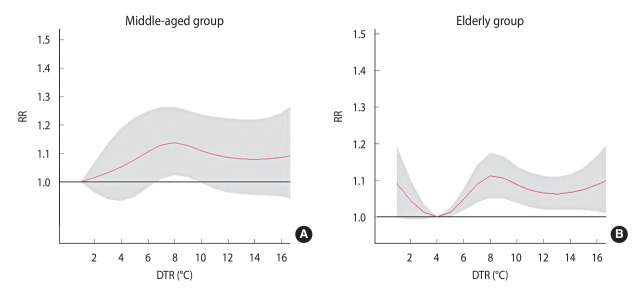
Cumulative effect of diurnal temperature range (DTR) on intracerebral hemorrhage admissions in (A) middle-aged group and (B) elderly group. RR, relative risk.

Figure 2 illustrates the overall impact of DTR on ICH among the middle-aged and elderly populations, suggesting that a higher DTR may elevate the risk of hospitalization for ICH in both age groups. Notably, in the elderly group, the risk of ICH decreased as DTR increased when the range was within 4°C.

**Figure 3.**
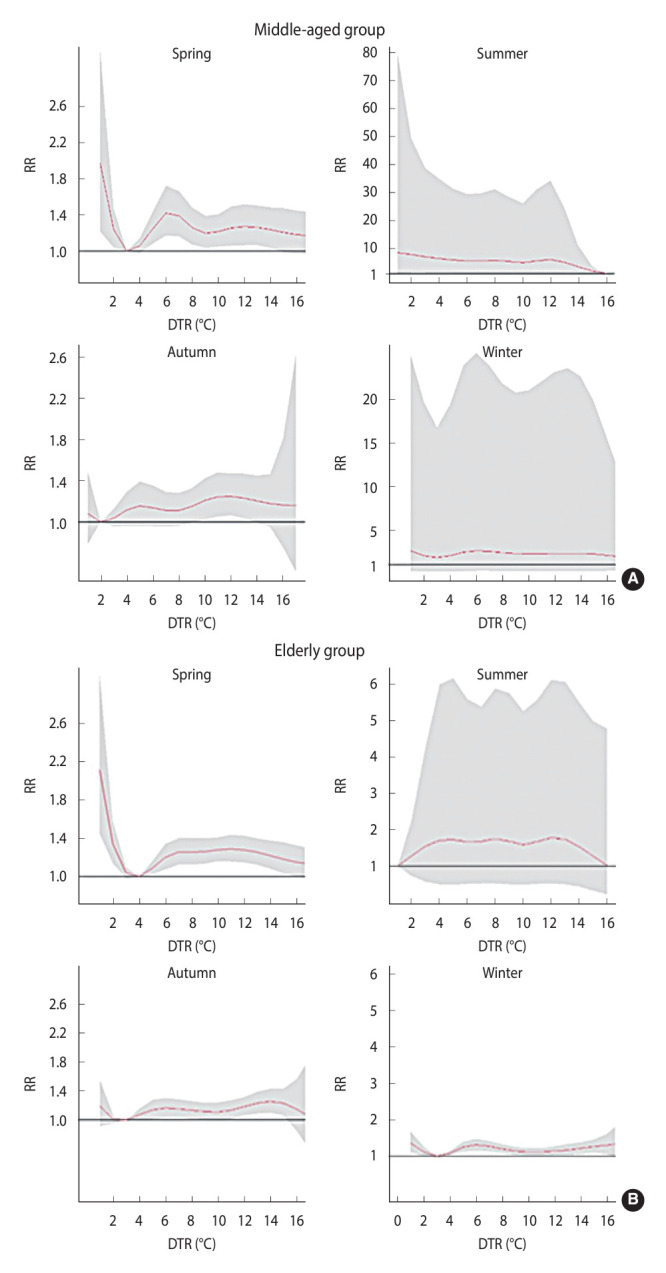
Cumulative effect of diurnal temperature range (DTR) on intracerebral hemorrhage admissions in different seasons of (A) middle-aged group and (B) elderly group. RR, relative risk.

Table 3 and Figure 3 illustrate the cumulative impact of DTR on ICH hospitalization among middle-aged and elderly populations in different seasons. Table 3 reveals that during spring, a higher DTR was associated with a greater risk of hospitalization for both middle-aged and elderly people. When the DTR was low (less than 3°C for middle-aged and less than 4°C for elderly individuals), both age groups also faced an elevated risk of hospitalization. In the summer, a low DTR was associated with a higher risk of ICH hospitalization for middle-aged individuals. Conversely, in autumn, a high DTR was linked to a greater risk of ICH hospitalization for both middle-aged and elderly populations. During winter, the risk of ICH hospitalization in the elderly rose regardless of whether the temperature difference was high (greater than 3°C) or low (less than 3°C).

Table 4 presents the cumulative lag effects of extreme DTR on hospitalization for ICH in middle-aged individuals, categorized by season. The P_2.5_ and P_97.5_ values represent extremely low and extremely high DTR values, respectively. In the spring, only the extremely low DTR exhibited a lag effect in this population. The initial effect persisted until lag0-4 days and subsided; the effect then reemerged at lag0-11 (RR, 1.21; 95% CI, 1.02 to 1.48), with a peak at lag0-12 (RR, 1.32; 95% CI, 1.11 to 1.42). During the summer, both extremely high and extremely low DTR began to influence hospitalizations starting at lag0-14. No lag effects of extreme DTR were observed in autumn or winter. Overall, middle-aged individuals should be aware of the lag effect occurring approximately 14 days after extreme DTR in the summer and about 11 days following extremely low DTR in the spring.

Table 4 presents the cumulative lag effect of extreme DTR on ICH hospitalization among the elderly, with P_2.5_ and P_97.5_ again representing extremely low and extremely high DTR, respectively. In spring, the impact of extremely low DTR began to appear at lag0-5 days (RR, 1.20; 95% CI, 1.01 to 1.36), and continued to increase until lag0-14. In comparison, the influence of extremely high DTR diminished as the number of lag days increased, disappearing by lag0-9 (RR, 1.23; 95% CI, 0.99 to 1.38). Regarding summer, the lag effects of both extremely low and extremely high DTR were statistically insignificant. In autumn, the effect of an extremely high DTR was observed at lag0-14. In winter, the impact of an extremely high DTR began at lag0-1 (RR, 1.21; 95% CI, 1.04 to 1.42) and grew until lag0-10 (RR, 1.39; 95% CI, 1.04 to 1.64), exhibiting a peak before declining. The effect of extremely low DTR started at lag0-4 and persisted until lag0-14. Overall, among the elderly, the heightened risk of ICH associated with DTR in the spring and winter manifested rapidly and lasted for approximately 1 week to 2 weeks.

The main results of the sensitivity analyses remained robust when we adjusted the df for DTR from 3 to 5 (Supplementary Material 7). Consistent findings were also observed when we varied the maximum lag for DTR from 14 days to 21 days (Supplementary Material 8) and when we modified the times of admission (Supplementary Material 9).

## DISCUSSION

This study presents a comprehensive analysis of the effects of daily fluctuations in DTR on hospitalizations for ICH. Our research is unique in that it centers on DTR as the primary exposure, considering its interplay with seasonal temperatures and variables such as social factors among middle-aged and elderly populations in central China. The findings of this study could be instrumental in the prevention and control of ICH.

We observed that the most prominent difference in hospitalizations occurred between December 31 and January 1. This discrepancy may stem from the annual settlement of medical insurance and the impact of holidays at the end of the year, which could influence reporting practices. Consequently, hospitalizations on January 1 were markedly higher than those on December 31. Our findings also indicate that the effects of DTR on ICH hospitalizations in the middle-aged demographic are predominantly delayed, manifesting after an extended period (> 10 days), except for low DTR during spring (< 2°C). The lag in the impact of DTR on ICH onset may be attributed to the time required for certain high-risk factors to develop or change [35]. The risk incrementally increases with rising DTR in spring and autumn, suggesting that the body’s thermoregulatory system may struggle to swiftly adapt to these environmental shifts [33]. In spring, a low DTR (< 3°C) coupled with sustained low temperatures provoked a rapid increase in the risk of ICH hospitalization among middle-aged individuals. Similarly, a persistent high-temperature environment with low DTR during summer elevated the risk of hospitalization for ICH in this age group. Potential underlying mechanisms include elevated blood pressure, peripheral vasoconstriction and dehydration, changes in salt intake, and heightened blood viscosity due to continuous exposure to extreme temperatures, whether low or high [34,38]. This implies that comprehensive health education on ICH, along with timely weather forecasts that highlight DTR, could effectively mitigate the risk of brain hemorrhage [34]. Additionally, the establishment of a high-temperature health alert system, greater utilization of air conditioning, avoidance of prolonged outdoor labor, and vigilant monitoring of health status may reduce the likelihood of hospitalization for ICH in middle-aged individuals [44].

The impact of DTR on ICH hospitalizations in the elderly resembled that in the middle-aged group in spring, but the effect manifested more quickly after exposure. The diminished thermoregulatory function in elderly people makes them particularly susceptible to temperature fluctuations, and the higher prevalence of chronic diseases in this population may further increase their sensitivity. Consequently, the risks associated with DTR manifested more rapidly in the elderly than in the middle-aged group [27,38]. Early warning systems and medical services must offer convenient access and provide prompt support for the elderly. This population should also be vigilant about staying warm to prevent exposure to sustained low temperatures in spring and winter. Additionally, caregivers should monitor weather forecasts and promptly assist elderly individuals to mitigate their risk [14]. In autumn, a high DTR may increase the risk of ICH hospitalization among the elderly. Therefore, among older adults, it is crucial to wear proper clothing, limit time spent outdoors, and maintain a healthy living environment with appropriate temperatures to reduce the risk of ICH [2].

This study has several limitations. First, the exact time of ICH onset prior to hospitalization was unknown, which means that using hospital admission as the primary outcome could result in an inaccurate assessment of the impact of DTR. However, patients with ICH are usually admitted to the hospital immediately for treatment. The median time from onset to admission has been reported to be 6.25 hours, with an interquartile range of 2.5 hours to 24.0 hours [47]. Additionally, individuals with mild or asymptomatic ICH may not always seek hospitalization, potentially leading to an underestimation of the true incidence of ICH. Despite this, ICH is responsible for a high proportion of stroke-related mortality and morbidity, and few demonstrably effective treatments are available for its acute management or prevention. The proportion of severe admissions is much higher than that of mild or asymptomatic admissions. Therefore, hospitalization is a reasonable proxy for estimating incidence based on the available data [49,50]. Second, the environmental exposure measurement used in this study is an average estimate for the local population and does not account for individual variations in exposure. This limitation hampers the ability to control for individual-level confounding factors, indicating a need for further research. Finally, the ecological nature of this study means that its findings cannot be used to establish a causal relationship.

After adjusting for other confounding factors, we observed a distinct non-linear relationship between DTR and hospitalization for ICH among middle-aged and elderly populations. The influence of DTR on ICH hospitalization risk varied significantly across seasons and between age groups, with the elderly participants demonstrating greater sensitivity to DTR. We suggest that weather forecasting services should report and highlight DTR values, and targeted interventions should be developed for populations that are particularly vulnerable to these changes.

## Figures and Tables

**Figure f4-epih-46-e2024053:**
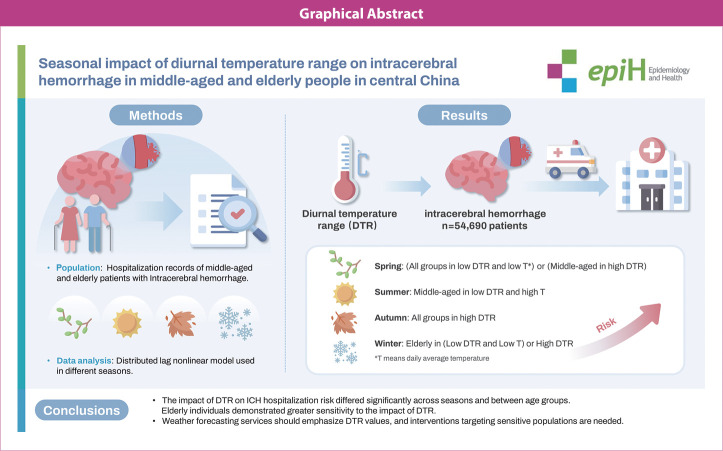


**Table 1.** Distribution of daily admissions of middle-aged and elderly patients with intracerebral hemorrhage in Hunan Province in 2019 (person-times)

Group	Total population^1^	Admissions^2^	X_min_	Percentile^3^			X_max_
				P_25_	P_50_	P_75_	
Male	11,514,871 (50.9)	34,383 (62.9)	12	80	95	123	211
Female	11,105,904 (49.1)	20,307 (37.1)	5	46	60	71	111
Middle-aged	13,064,922 (57.8)	16,824 (29.8)	7	39	48	58	102
Elderly	9,555,853 (42.2)	37,866 (70.2)	10	89	107	137	220
Total	22,620,775	54,690	17	130	154	195	322

Values are presented as number (%). min, minimum; max, maximum.

1 The proportion of various population groups relative to the total population of Hunan Province in 2019.

2 The proportion of admissions of various groups relative to total hospital admissions in Hunan Province in 2019.

3 Percentile refers to the daily distribution of admissions.

**Table 2.** Monthly distribution of admissions for intracerebral hemorrhage among middle-aged and elderly people in Hunan Province in 2019 (person-times)

Month	Admissions of middle-aged patients	Admissions of elderly patients
Jan	1,946 (11.6)	4,398 (11.6)
Feb	1,511 (9.0)	2,896 (7.6)
Mar	1,826 (10.8)	4,116 (10.9)
Apr	1,433 (8.5)	3,085 (8.1)
May	1,266 (7.5)	3,114 (8.2)
Jun	1,112 (6.6)	2,474 (6.5)
Jul	1,112 (6.6)	2,551 (6.7)
Aug	1,071 (6.4)	2,244 (5.9)
Sep	1,216 (7.2)	2,644 (7.0)
Oct	1,365 (8.1)	3,197 (8.4)
Nov	1,686 (10.0)	4,077 (10.8)
Dec	1,280 (7.6)	3,070 (8.1)
Total	16,824 (100)	37,866 (100)

Values are presented as number (%).

**Table 3.** Cumulative effect of diurnal temperature range (DTR) on admissions for intracerebral hemorrhage by season

DTR/°C	Spring	Summer	Autumn	Winter
Middle-aged reference^1^	3°C	16°C	2°C	22°C
1	1.97 (1.21, 3.20)^*^	8.44 (0.90, 79.06)	1.08 (0.78, 1.48)	2.61 (0.27, 25.01)
3	1.00 (1.00, 1.00)	7.04 (1.28, 38.74)^*^	1.04 (0.95, 1.13)	1.80 (0.19, 16.78)
5	1.25 (1.08, 1.44)^*^	5.86 (1.09, 31.44)^*^	1.15 (0.96, 1.39)	2.44 (0.25, 23.87)
10	1.21 (1.04, 1.41)^*^	4.91 (0.92, 26.11)	1.21 (1.02, 1.43)^*^	2.25 (0.24, 21.04)
15	1.21 (0.99, 1.47)	1.95 (1.04, 3.67)^*^	1.18 (0.95, 1.47)	2.21 (0.25, 19.77)
Elderly reference^1^	4°C	1°C	3°C	3°C
1	2.12 (1.44, 3.11)^*^	1.00 (1.00, 1.00)	1.19 (0.92, 1.54)	1.37 (1.11, 1.69)^*^
3	1.04 (0.98, 1.10)	1.54 (0.56, 4.27)	1.00 (1.00, 1.00)	1.00 (1.00, 1.00)
5	1.09 (1.02, 1.16)^*^	1.72 (0.48, 6.17)	1.41 (1.02, 1.28)^*^	1.25 (1.12, 1.40)^*^
10	1.28 (1.16, 1.41)^*^	1.58 (0.48, 5.27)	1.11 (0.99, 1.25)	1.12 (1.02, 1.23)^*^
15	1.18 (1.03, 1.36)^*^	1.26 (0.32, 4.99)	1.23 (1.06, 1.43)^*^	1.26 (1.09, 1.46)^*^

Values are presented as relative risk (95% confidence interval).

1 The value of relative risk was estimated by considering the DTR with the lowest risk as the reference group.

* p<0.05.

**Table 4.** Cumulative lag effect of extreme diurnal temperature range (DTR) on admissions for intracerebral hemorrhage in elderly people by season

Lag (day)	Spring	Summer	Autumn	Winter
Reference^1^	4°C	1°C	3°C	3°C
2.5% DTR	2°C	4°C	2°C	1°C
0	1.09 (0.89, 1.33)	0.58 (0.25, 1.33)	0.98 (0.92, 1.05)	1.19 (0.99, 1.41)
0-4	1.18 (0.99, 1.42)	2.11 (0.78, 5.73)	0.99 (0.93, 1.05)	1.34 (1.11, 1.62)^*^
0-7	1.33 (1.10, 1.60)^*^	2.33 (0.78, 6.94)	0.97 (0.91, 1.04)	1.37 (1.12, 1.67)^*^
0-14	1.34 (1.14, 1.59)^*^	1.71 (0.49, 5.99)	1.01 (0.95, 1.07)	1.37 (1.11, 1.69)^*^
97.5% DTR	17°C	12°C	15°C	14°C
0	1.61 (1.21, 2.14)^*^	0.33 (0.14, 0.88)	1.23 (0.94, 1.61)	1.06 (0.81, 1.39)
0-4	1.44 (1.16, 1.78)^*^	2.50 (0.92, 6.82)	0.98 (0.79, 1.21)	1.26 (1.02, 1.55)^*^
0-7	1.47 (1.18, 1.82)^*^	2.74 (0.92, 8.20)	1.01 (0.81, 1.25)	1.31 (1.07, 1.61)^*^
0-14	1.13 (0.99, 1.30)	1.78 (0.52, 6.12)	1.23 (1.06, 1.43)^*^	1.22 (1.07, 1.39)^*^

Values are presented as relative risk (95% confidence interval).

1 The value of relative risk was estimated by considering the DTR with the lowest risk as the reference group.

* p<0.05.

## Supplementary materials

Supplementary Material 1. The geographic distribution of the secondary and tertiary hospitals.

Supplementary Material 2. Daily meteorological and air quality data of 122 districts/counties in 2019 Hunan

Supplementary Material 3. Spearman correlation coefficients of daily exposure variables

Supplementary Material 4. The average length and their range of ICH hospital stays of middle-aged and elderly admissions in 2019 Hunan (days)

Supplementary Material 5. Time series plot of ICH admissions for middle-aged and elderly people in 2019 Hunan.

Supplementary Material 6. Distribution of monthly mean temperature and diurnal temperature range (DTR) in 2019 Hunan.

Supplementary Material 7. Sensitivity analyses of the effects of diurnal temperature range (DTR) effects on intracerebral hemorrhage admissions of middle-aged group and elderly group, changing df for diurnal temperature range.

Supplementary Material 8. Sensitivity analyses of the effects of diurnal temperature range (DTR) on intracerebral hemorrhage admission of middle-aged group and elderly group, changing maximum lag days for diurnal temperature range.

Supplementary Material 9. Sensitivity analyses of the effects of diurnal temperature range on Intracerebral hemorrhage admission of middle-aged group and elderly group, changing the admission times.
