# Supplementary material for: Seasonal impact of diurnal temperature range on intracerebral hemorrhage in middle-aged and elderly people in central China
Source: Epidemiol Health. 2024 Jun 11;46:e2024053. doi: 10.4178/epih.e2024053 (PMC11573486; doi:10.4178/epih.e2024053)

Supplementary materials

**Supplementary Material 1.** The geographic distribution of the secondary and tertiary hospitals.


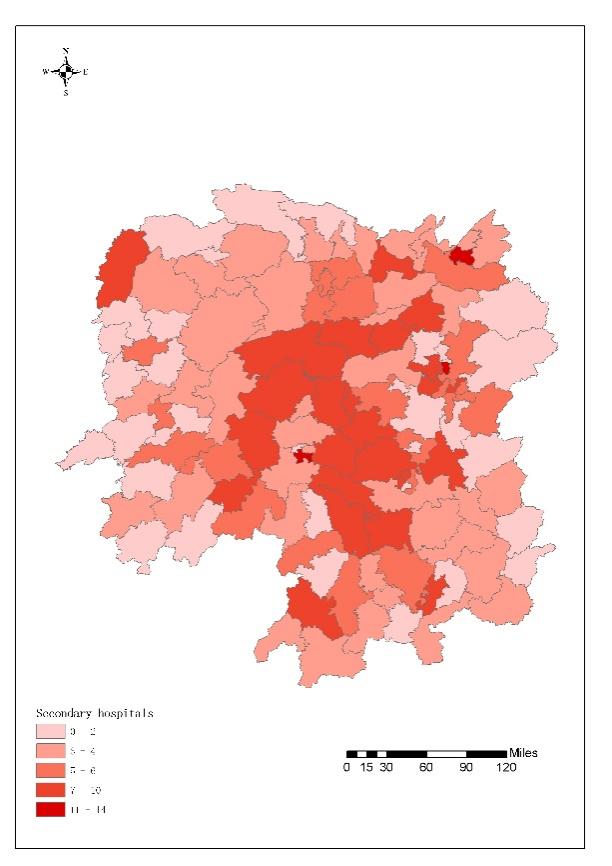

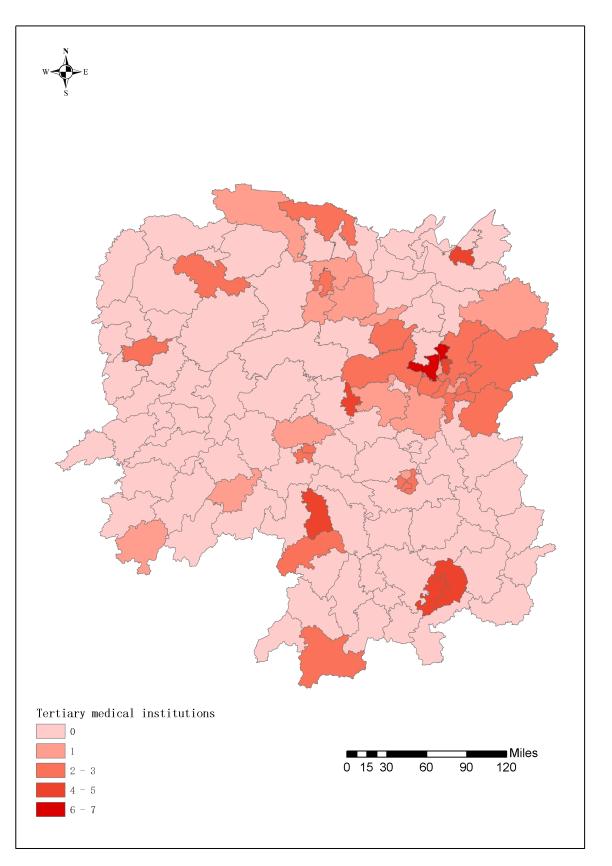

Supplement: Supplementary Material 1. — The geographic distribution of the secondary and tertiary hospitals. [file epih-46-e2024053-Supplementary-1.docx]
