# Supplementary material for: Seasonal impact of diurnal temperature range on intracerebral hemorrhage in middle-aged and elderly people in central China
Source: Epidemiol Health. 2024 Jun 11;46:e2024053. doi: 10.4178/epih.e2024053 (PMC11573486; doi:10.4178/epih.e2024053)
Supplement: Supplementary Material 2. — Daily meteorological and air quality data of 122 districts/counties in 2019 Hunan [file epih-46-e2024053-Supplementary-2.docx]

**Supplementary Material 2.** Daily meteorological and air quality data of 122 districts/counties in 2019 Hunan

| Variables | *X*_min_ | *P*_25_ | *P*_50_ | *P_75_* | *X*_max_ | *QR* |
| --- | --- | --- | --- | --- | --- | --- |
| Average temperature(℃) | -5.5 | 11.3 | 19.6 | 26.7 | 33.4 | 15.4 |
| Min temperature(℃) | -7.6 | 7.8 | 15.3 | 22.8 | 30.1 | 15.0 |
| Max temperature（℃） | -4.6 | 14.4 | 24.1 | 30.6 | 38.6 | 16.2 |
| Relative humidity（%） | 39.7 | 76.0 | 81.2 | 86.3 | 99.6 | 10.3 |
| DTR(℃) | 0.004 | 4.8 | 7.7 | 10.1 | 22.3 | 5.3 |
| PM_2.5_(μg/m^3^) | 1.8 | 21.4 | 31.2 | 46.5 | 288.0 | 25.1 |
| PM_10_(μg/m^3^） | 6.9 | 38.2 | 52.2 | 75.2 | 363.0 | 37.0 |
| SO_2_(μg/m^3^） | 1.4 | 6.7 | 9.1 | 11.9 | 48.5 | 5.2 |
| NO_2_(μg/m^3^) | 1.6 | 13.1 | 17.8 | 25.1 | 100.3 | 12.0 |
| O_3_(μg/m^3^） | 4.0 | 64.8 | 94.7 | 123.1 | 250.4 | 58.3 |
| CO(mg/m^3^） | 0.1 | 0.7 | 0.9 | 1.0 | 2.8 | 0.3 |

DTR indicates diurnal temperature range; PM_2.5_ indicates fine particulate matter; PM_10_ indicates inhalable particulate matter; SO_2_ indicates sulfur dioxide; NO_2_ indicates nitrogen dioxide; O_3_ indicates ozone; CO indicates carbon monoxide; QR, indicates interquartile range.
