# Supplementary material for: Seasonal impact of diurnal temperature range on intracerebral hemorrhage in middle-aged and elderly people in central China
Source: Epidemiol Health. 2024 Jun 11;46:e2024053. doi: 10.4178/epih.e2024053 (PMC11573486; doi:10.4178/epih.e2024053)
Supplement: Supplementary Material 3. — Spearman correlation coefficients of daily exposure variables [file epih-46-e2024053-Supplementary-3.docx]

**Supplementary Material 3.** Spearman correlation coefficients of daily exposure variables

| Variables | Average temperature | Relative humidity | DTR | PM_2.5_ | PM_10_ | SO_2_ | NO_2_ | O_3_ | CO |
| --- | --- | --- | --- | --- | --- | --- | --- | --- | --- |
| Average temperature (℃) | — | — | — | — | — | — | — | — | — |
| Relative humidity (%) | 0.22* | — | — | — | — | — | — | — | — |
| DTR(℃) | 0.34* | 0.01* | — | — | — | — | — | — | — |
| PM_2.5_(μg/m^3^) | -0.15* | -0.06* | -0.08* | — | — | — | — | — | — |
| PM_10_(μg/m^3^） | -0.19* | -0.24* | -0.10* | 0.83* | — | — | — | — | — |
| SO_2_(μg/m^3^） | -0.13* | -0.31* | -0.07* | 0.29* | 0.37* | — | — | — | — |
| NO_2_(μg/m^3^) | 0.01 | -0.10* | -0.05* | 0.57* | 0.55* | 0.48* | — | — | — |
| O_3_(μg/m^3^） | -0.07 | 0.01 | 0.03 | -0.19* | -0.08* | -0.06* | -0.20* | — | — |
| CO(mg/m^3^） | -0.05* | -0.22* | -0.05* | 0.35* | 0.35* | 0.38* | 0.29* | -0.18* | — |

DTR indicates diurnal temperature range; PM_2.5_ indicates fine particulate matter; PM_10_ indicates inhalable particulate matter; SO_2_ indicates sulfur dioxide; NO_2_ indicates nitrogen dioxide; O_3_ indicates ozone; CO indicates carbon monoxide.

*p*<*0.05.
