# Supplementary material for: Seasonal impact of diurnal temperature range on intracerebral hemorrhage in middle-aged and elderly people in central China
Source: Epidemiol Health. 2024 Jun 11;46:e2024053. doi: 10.4178/epih.e2024053 (PMC11573486; doi:10.4178/epih.e2024053)
Supplement: Supplementary Material 4. — The average length and their range of ICH hospital stays of middle-aged and elderly admissions in 2019 Hunan (days) [file epih-46-e2024053-Supplementary-4.docx]

**Supplementary Material 4.** The average length and their range of ICH hospital stays of middle-aged and elderly admissions in 2019 Hunan (days)

| Variables | Median Hospital stays in different seasons | | | | Total median length |
| --- | --- | --- | --- | --- | --- |
|  | Spring | Summer | Autumn | Winter |  |
| Male | 21(17-23) | 14(11-16) | 20(17-25) | 27(23-31) | 21(14-31) |
| Female | 16(14-20) | 14(12-15) | 16(13-18) | 24(21-26) | 17(15-24) |
| middle-aged | 24(20-27) | 16(14-19) | 18(14-21) | 20(13-22) | 19(17-23) |
| elderly | 28(25-33) | 15(13-20) | 21(18-23) | 30(25-31) | 24(20-28) |
| Total | 20(16-27) | 15(11-20) | 18(14-24) | 23(20-28) | 19(14-23) |
