# Supplementary material for: Seasonal impact of diurnal temperature range on intracerebral hemorrhage in middle-aged and elderly people in central China
Source: Epidemiol Health. 2024 Jun 11;46:e2024053. doi: 10.4178/epih.e2024053 (PMC11573486; doi:10.4178/epih.e2024053)
Supplement: Supplementary Material 6. — Distribution of monthly mean temperature and diurnal temperature range (DTR) in 2019 Hunan. [file epih-46-e2024053-Supplementary-6.docx]

**
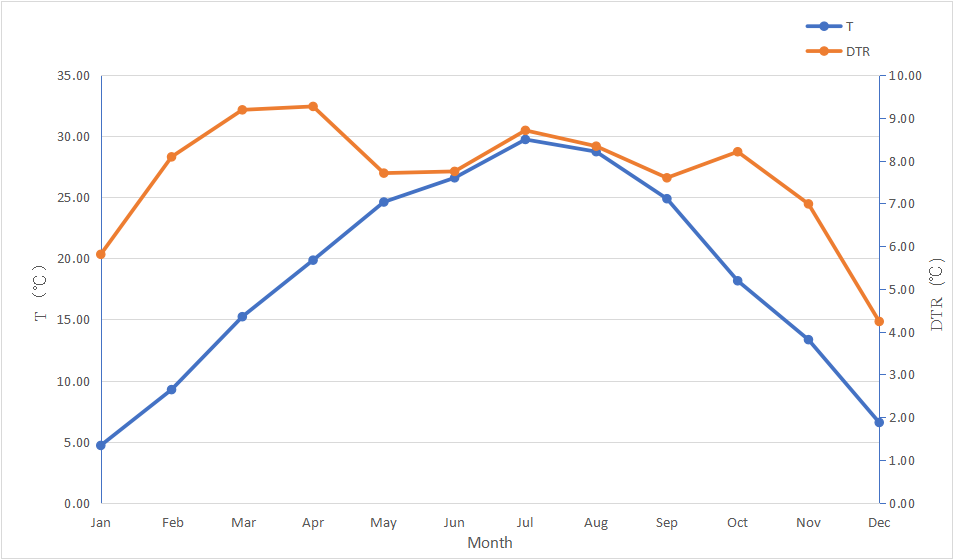
**

**Supplementary Material 6.** Distribution of monthly mean temperature and diurnal temperature range (DTR) in 2019 Hunan.
