# Supplementary material for: Seasonal impact of diurnal temperature range on intracerebral hemorrhage in middle-aged and elderly people in central China
Source: Epidemiol Health. 2024 Jun 11;46:e2024053. doi: 10.4178/epih.e2024053 (PMC11573486; doi:10.4178/epih.e2024053)
Supplement: Supplementary Material 8. — Sensitivity analyses of the effects of diurnal temperature range (DTR) on intracerebral hemorrhage admission of middle-aged group and elderly group, changing maximum lag days for diurnal temperature range. [file epih-46-e2024053-Supplementary-8.docx]

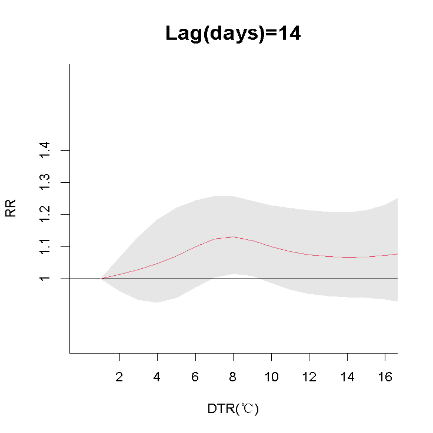

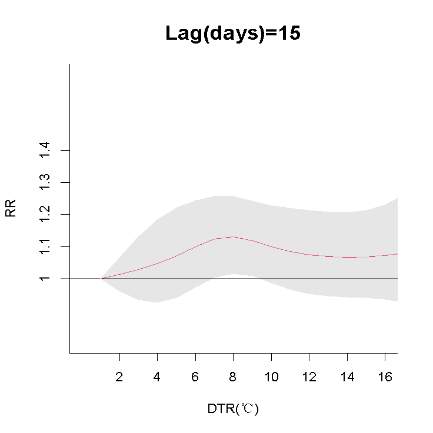

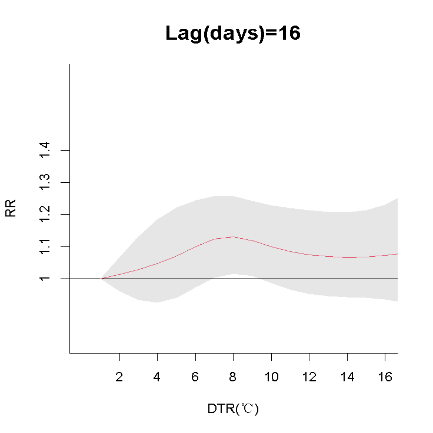

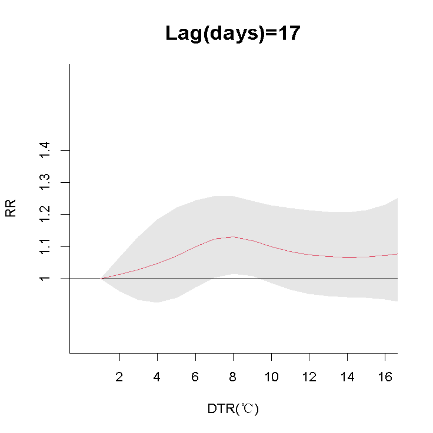

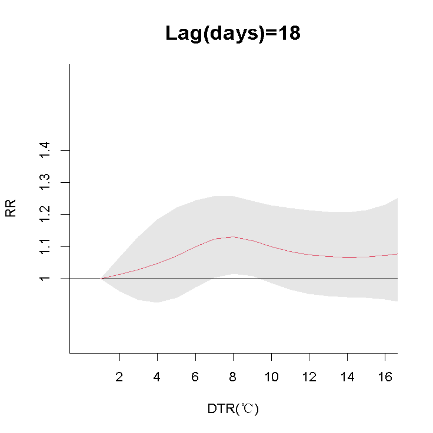

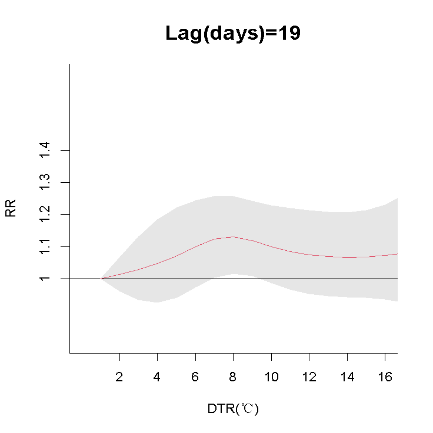

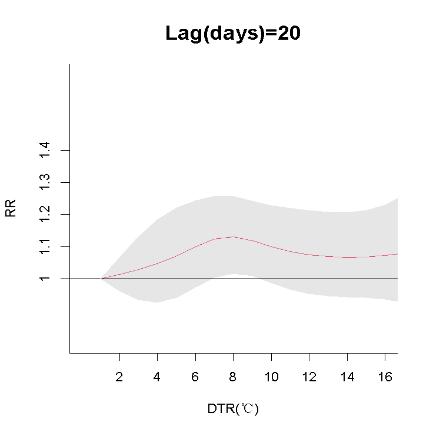

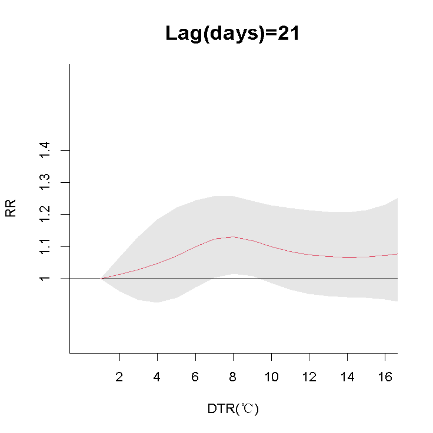


**Middle-aged group**


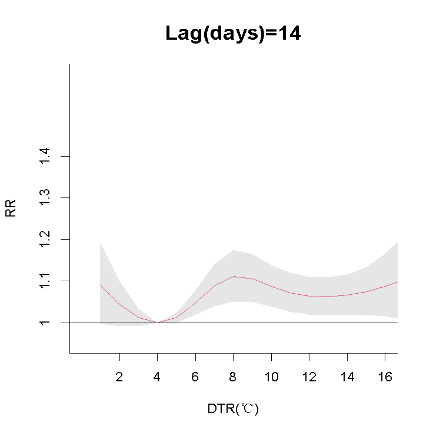

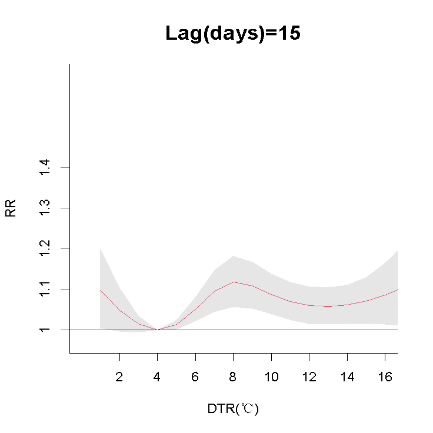

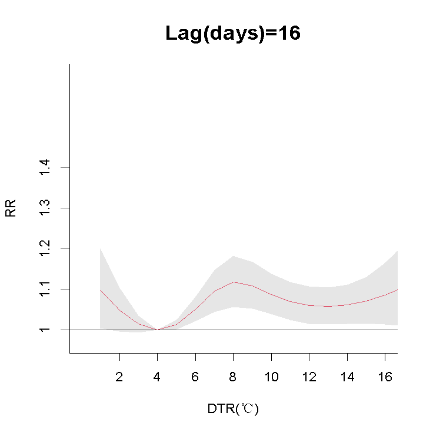

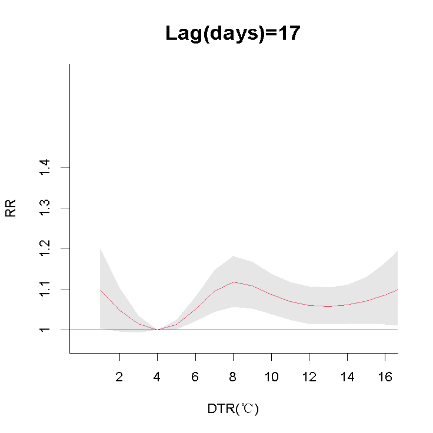

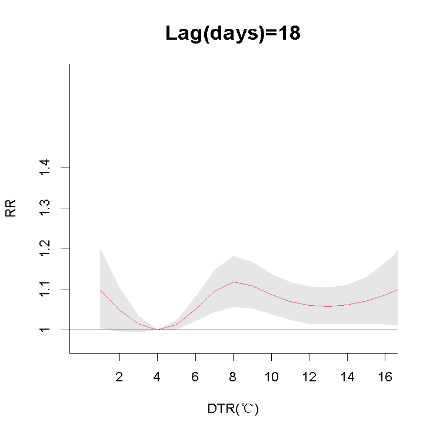

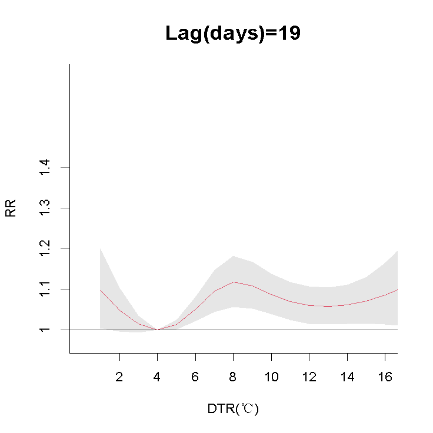

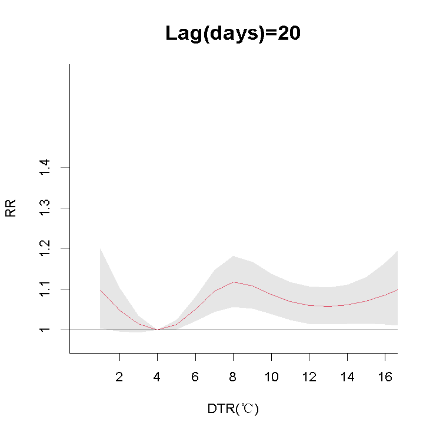

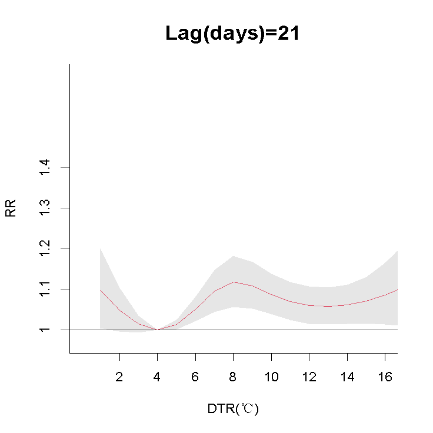


**Elderly group**

**Supplementary Material 8.** Sensitivity analyses of the effects of diurnal temperature range (DTR) on intracerebral hemorrhage admission of middle-aged group and elderly group, changing maximum lag days for diurnal temperature range.
