# Supplementary material for: Seasonal impact of diurnal temperature range on intracerebral hemorrhage in middle-aged and elderly people in central China
Source: Epidemiol Health. 2024 Jun 11;46:e2024053. doi: 10.4178/epih.e2024053 (PMC11573486; doi:10.4178/epih.e2024053)
Supplement: Supplementary Material 9. — Sensitivity analyses of the effects of diurnal temperature range on Intracerebral hemorrhage admission of middle-aged group and elderly group, changing the admission times. [file epih-46-e2024053-Supplementary-9.docx]

**
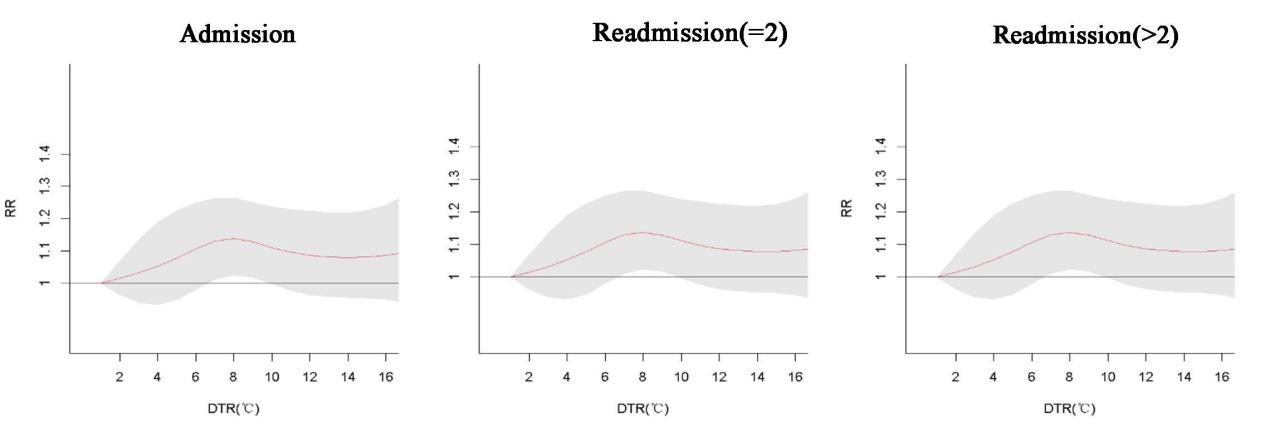

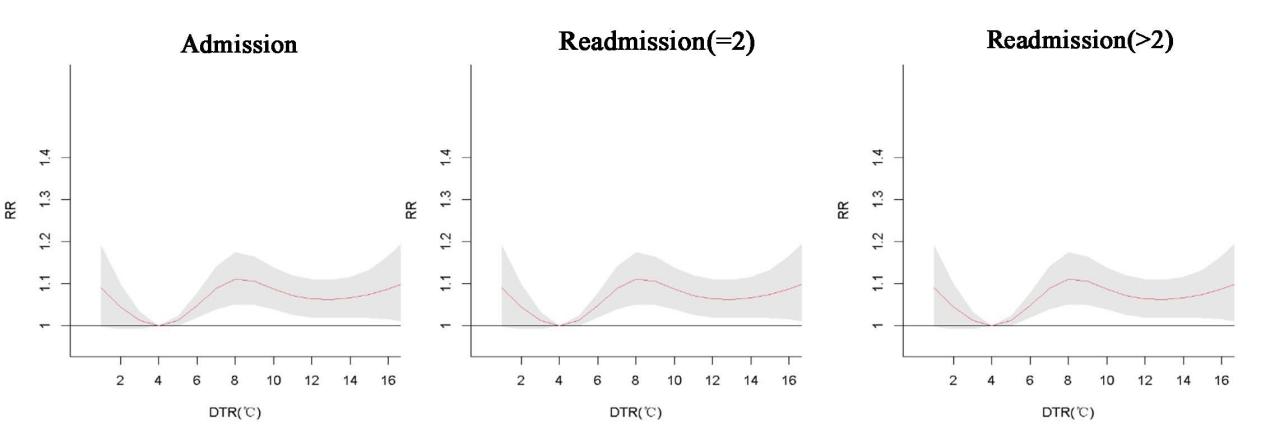
**

**Elderly group**

**Middle-aged group**

**Supplementary Material 9.** Sensitivity analyses of the effects of diurnal temperature range on Intracerebral hemorrhage admission of middle-aged group and elderly group, changing the admission times.
